# Time trends in stroke incidence and in prevalence of risk factors in Southern Germany, 1989 to 2008/09

**DOI:** 10.1038/s41598-018-30350-8

**Published:** 2018-08-10

**Authors:** Inke Thiele, Jakob Linseisen, Margit Heier, Rolf Holle, Inge Kirchberger, Annette Peters, Barbara Thorand, Christa Meisinger

**Affiliations:** 1 Institute of Epidemiology, Helmholtz Zentrum München, German Research Center for Environmental Health, Neuherberg, Germany; 2 Independent Research Group Clinical Epidemiology, Helmholtz Zentrum München, German Research Center for Environmental Health, Neuherberg, Germany; 3 Chair of Epidemiology, Ludwig-Maximilians Universität München, UNIKA-T Augsburg, Germany; 4 Institute of Health Economics and Health Care Management, Helmholtz Zentrum München, German Research Center for Environmental Health, Neuherberg, Germany

## Abstract

In prior studies, stroke incidence has mainly shown either declining time trends or stable rates in high-income countries. Changes could partially be linked to trends in classic cardiovascular disease (CVD) risk factors. In the present study, we analyzed the incidence of stroke in parallel with the prevalence of CVD risk factors over time in a German population. Data from three independent population-based MONICA/KORA Augsburg surveys conducted in 1989/90 (S2), 1994/95 (S3), and 1999/2001 (S4) were used to calculate age-standardized incidence rates (IR) of first-ever stroke over eight years from each baseline survey. Furthermore, the age-standardized prevalence rates of CVD risk factors were analyzed for these surveys. Changes in IR or prevalence were considered significantly different if their 95% confidence intervals (CI) did not overlap. The age-standardized IR of stroke showed no significant time trend (S2: IR = 203.4 per 100,000 person-years; CI 176.4–233.4, S3: IR = 225.6; 197.1–257.0, S4: IR = 209.9; CI 182.4–240.3). In agreement, the prevalence of the CVD risk factors was quite stable over time, showing divergent, but mostly non-significant changes. However, due to the aging Western societies and the longer survival time of stroke patients, the total number of stroke patients in the population will increase even with a stable IR.

## Introduction

Stroke is still one of the leading causes of death and disability worldwide^1,2^. The burden of ischemic and hemorrhagic stroke increased significantly worldwide between 1990 and 2010 in terms of the absolute number of incident events, number of deaths, and disability-adjusted life-years (DALYs) lost^3^. While incidence rates of stroke increased in low- and middle-income countries^3,4^, they remained stable^5,6^ or declined in high-income countries^3,4,7^. In 2010, for example, the age-standardized incidence of ischemic stroke per 100 000 person-years ranged from 52 in Qatar to 434 in Lithuania^3^.

Knowledge about cardiovascular disease (CVD) risk factors was originally derived mainly from studies in European populations^8^. Several potential risk factors for CVD were identified in these studies as causal factors, including tobacco smoking, high blood pressure and obesity^8^. Recently, the Global Burden of Disease Study 2013^9^ and the case-control study INTERSTROKE 2007–2015^10^ observed that 90% of the stroke burden is attributable to modifiable risk factors. In the INTERSTROKE study, 10 modifiable risk factors were identified, namely hypertension, smoking, diabetes mellitus, physical inactivity, poor diet, unfavourable psychosocial factors, abdominal obesity, alcohol abuse, cardiac diseases, and high apolipoprotein ApoB/ApoA1 ratio^10^. In addition, it was found that hypertension is twice as important for the manifestation of stroke as for coronary heart disease^11^.

Several studies identified and quantified a linkage between the time trend in stroke incidence and the temporal changes in classic CVD risk factors: In the populations of the World Health Organization Monitoring of Trends and Determinants in Cardiovascular Diseases (MONICA) project, 38% of the variation in stroke events in women (2% in men) was explained by changes in systolic blood pressure^7^. In men, most of the variation was explained by total cholesterol (10%). In the Tromsø Study, Norway, classic CVD risk factors explained 57% of the decline in ischemic stroke incidence from 1995 to 2012^6^. The reduction in systolic BP was the largest single contributor, accounting for 26% of the observed decline.

In the present investigation, trends in the incidence of stroke, as well as concomitantly the prevalence of classic CVD risk factors in Southern Germany were analyzed using data of the three independent representative MONICA/KORA Augsburg surveys conducted between 1989/90 and 1999/2001 and followed-up until 2008/09.

## Methods

### Study population

To estimate the prevalence and distribution of cardiovascular risk factors in the MONICA project in the study region of Augsburg, independent cross-sectional surveys were conducted in 1984/85 (S1), 1989/90 (S2), and 1994/95 (S3). Between 1999 and 2001 (S4) a further cross-sectional survey was carried out in the framework of its regional successor project “Cooperative Health Research in the Region of Augsburg (KORA)”^12,13^. The study region was comprised of the city of Augsburg (Southern Germany) and its two surrounding counties.

**Figure 1:**
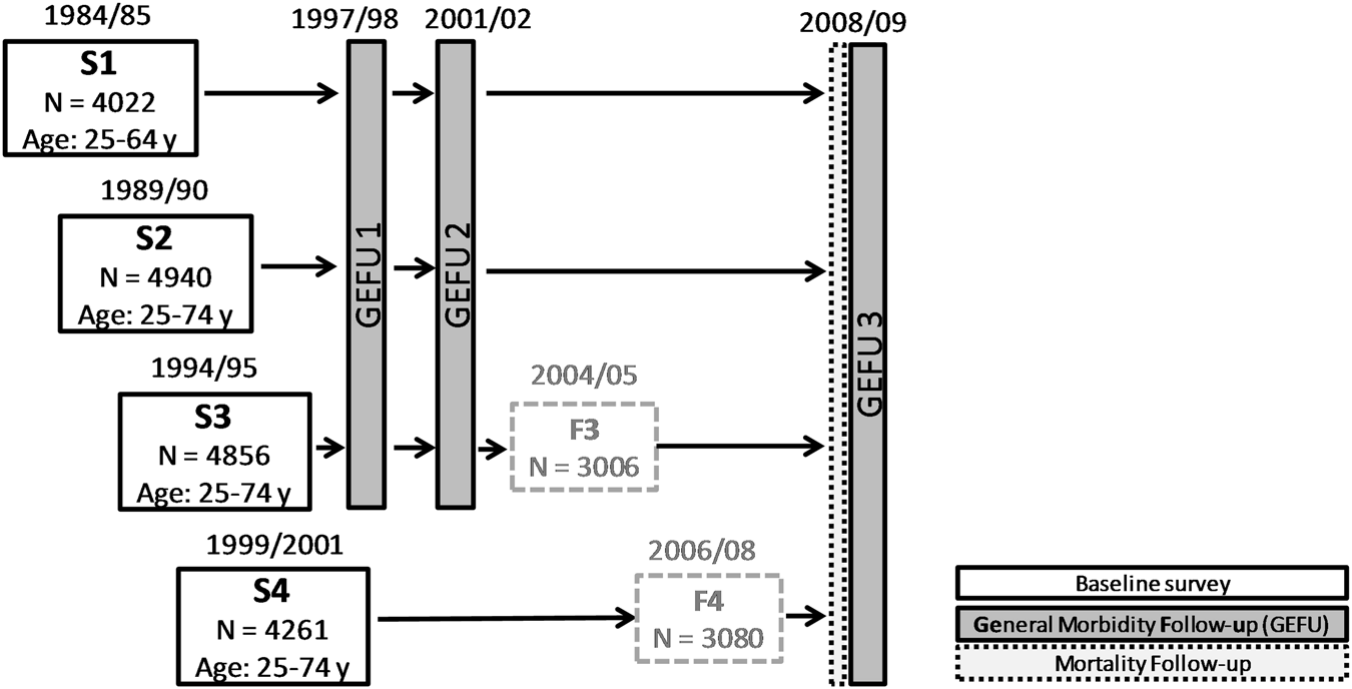
The MONICA/KORA cohort study: the four surveys S1–S4 and follow-up studies until the end of 2009.

As a stroke register has not been established in the region of Augsburg, information on incident stroke cases was gathered by following the participants of the four population-based surveys until 2008/09 (see Fig. 1) through regular postal questionnaires (GEFU 1, GEFU 2, GEFU 3) and mortality follow-ups. The studies were approved by the ethics committee of the Bavarian Medical Association. Written informed consent was obtained from each participant in accordance with institutional requirements and the Declaration of Helsinki Principles. All methods were performed in accordance with relevant guidelines and regulations.

Participants with any type of prevalent stroke at baseline, incident reversible stroke or undefined incident stroke as well as participants with missing data on prevalent or incident stroke, were excluded from the analyses. Thus, incident stroke was comprised of ischemic and hemorrhagic strokes.

The main analysis included participants from the MONICA/KORA surveys S2, S3, and S4, who all ranged in age from 25 to 74 years at baseline examination. However, the age range in S1 was limited to 25 to 64 years. As a result, participants from S1 were only included in a sub-analysis (see Table 1).

**Table 1:** Participants of the MONICA/KORA surveys included in the analyses.

	S1	S2	S3	S4
	1984/85	1989/90	1994/95	1999/2001
**Main analysis: participants aged 25–74 y (all types of stroke)**				
total N	—	4594	4384	3759
men n (%)	—	2315 (50.39)	2176 (49.64)	1825 (48.55)
women n (%)	—	2279 (49.61)	2208 (50.36)	1934 (51.45)
**Sub-analysis: participants aged 25–74 y; only ischemic stroke** ^**a**^				
total N	—	4577	4366	3747
men n (%)	—	2302 (50.29)	2166 (49.61)	1818 (48.52)
women n (%)	—	2275 (49.71)	2200 (50.39)	1929 (51.48)
**Sub-analysis: participants aged 25–64 y; only ischemic stroke** ^**a**^				
total N	3905	3703	3588	3129
men n (%)	1966 (50.35)	1840 (49.69)	1755 (48.91)	1502 (48.00)
women n (%)	1939 (49.65)	1863 (50.31)	1833 (51.09)	1627 (52.00)

^a^Participants with incident hemorrhagic stroke were excluded.

### Data collection and definition of variables

Non-fatal and fatal incident stroke cases during the follow-up period were included in the analyses. Eight years was the maximally consistent follow-up time for all surveys (see chapter “statistical analyses”). Non-fatal strokes were identified by postal follow-up questionnaires. Using data from participants’ hospital records and information gathered from their treating physicians, all self-reported potential incident stroke cases and the date of diagnosis were validated. To ascertain fatal strokes, the survival status of all participants was regularly checked using information provided by population registries inside and outside the study area. Death certificates of deceased participants, preserved by local health authorities, were analyzed and the main causes of death were extracted. Further information regarding fatal strokes was then obtained from the deceased’s hospital records. The same procedure was used to determine if an individual who died from a cause other than stroke had suffered a stroke in the time between the previous follow-up and the participant’s eventual death. The three-digit codes 430–434 of the International Classification of Diseases, Ninth Revision (ICD-9), German modification, were considered as death due to stroke.

Data on classic CVD risk factors were collected at baseline of each survey by trained medical staff using standardized interviews and medical examinations. Smokers were categorized into current/former/never smokers. “Current” was defined as smoking one or more cigarettes per day at baseline. Alcohol intake was dichotomized into high and moderate: High intake was defined as >20 g/day pure alcohol in men, and >10 g/day in women. Low leisure time physical activity was defined as ≤1 h/week of irregular sportive activity in summer and winter. Obesity was classified with a BMI ≥ 30 kg/m². Low education level was set as ≤12 y of school and apprenticeship or studies. A total cholesterol/high-density lipoprotein cholesterol ratio (TC/HDL) of  ≥5 was defined as elevated. Elevated uric acid was characterized as >420 µmol/l in men and >360 µmol/l in women. A diagnosis of diabetes mellitus and parents’ stroke history were self-reported. If the participants did not know if one or both of their parents had had a stroke (all kinds of stroke were included), it was counted as a “no”. As hypertension was assumed to be the main risk factor, we examined four different hypertension classifications, considering known and newly detected hypertension: “Actual hypertension” (blood pressure (BP) ≥ 140/90 mmHg and/or intake of antihypertensive medication, given that the subjects were aware of being hypertensive), “hypertension grade 2” (BP ≥ 160/100 mmHg), “hypertension grade 3” (BP ≥ 180/110 mmHg) and “use of antihypertensive medication”. Antihypertensive medication was defined according to the guidelines of the German hypertension league (Deutsche Hochdruckliga), which are based on the guidelines of the European Society of Hypertension, that were current at the time of each respective follow-up^14^. Blood pressure was measured three times in a sitting position after at least 5 minutes at rest with a random-zero sphygmomanometer in S1, S2, and S3 (Hawksley & Sons Ltd, Lancing, England) and by use of an oscillometric digital blood pressure monitor (HEM-705CP, Omron Corporation, Toky3o, Japan) in S4. The mean of the second and third measurement was calculated and used for the present analyses. Information on medication intake during the 7 days preceding the baseline examination was collected during the interview. Afterwards the medications were coded according to the “Rote Liste®”, the official German pharmaceutical catalog. In the S4 survey, medication data were collected using the standardized software IDOM (instrument for database-supported online registration of medication)^15^.

### Statistical analyses

As each survey had a different time of follow-up to GEFU3 (2008/09), the aging effect had to be taken into account. To examine if pure age-standardization is sufficient to compensate the aging effect, we computed the cumulative baseline hazard function (CUMHAZ) per 5 year intervals in unadjusted Cox proportional hazards regression models for S2 (1989/90–2008/09) and S3 (1994/95–2008/09). The CUMHAZ did not rise linearly. Therefore a time dependency of risk had to be assumed. For this reason, we used an equal follow-up time for each survey in the stroke trend analyses. As the shortest follow-up time from baseline to GEFU3 was eight years in the S4 survey, we used eight years as common follow-up time for all surveys.

Incidence rates of stroke were calculated per survey for the total study population as well as stratified by sex and age group. Afterwards, a direct age-standardization to the German population (population as of December 31, 2000) was performed. The 95% confidence intervals (CIs) were calculated considering the Poisson distribution. Differences in incidence rates were considered statistically significant if the 95% CIs did not overlap. A significant time trend exists if the change in the incidence rate between the first and the second survey, as well as between the second and the third survey and so on, was significant and pointed in the same direction.

We also performed stroke-trend sub-analyses where incident stroke included only ischemic stroke (hemorrhagic stroke was excluded). A further stroke-trend sub-analysis was conducted with data from all four surveys (S1–S4), restricted to participants aged 25 to 64 years at baseline. In order to analyze a longer consistent follow-up time, another sub-analysis was performed in participants of S2 and S3 using age-standardized incidence rates of ischemic stroke over 14 years of follow-up time.

The prevalence of the classic CVD risk factors were directly age-standardized to the German population as well. Afterwards the 5% Wald-type CIs of the prevalence rates were computed. Differences in prevalence were considered statistically significant if the 95% CIs did not overlap. These differences provide information on changes over time, as prevalence rates were derived from the baseline examinations, which were conducted at different points in time. All analyses were performed using the SAS software version 9.3 (SAS Institute, Inc., Cary, NC, USA).

### Data availability

The data that support the findings of this study are available from the Institute of Epidemiology, Helmholtz Zentrum München, German Research Center for Environmental Health (Neuherberg, Germany), but restrictions apply to the availability of these data, which were used under license for the current study, and so are not publicly available. Data are, however, available from the authors upon reasonable request and with permission of the Institute of Epidemiology, Helmholtz Zentrum München, German Research Center for Environmental Health.

## Results

The number of incident stroke cases and the age-standardized incidence rates from S2 to S4 (baseline age-range 25–74 y and eight years of follow-up) are shown in Table 2. The incidence rates in men were higher than in women. In the total sample, the age-standardized incidence rates from S2 to S4 were quite stable, and there was no significant time trend. A time trend was not observed when stratified by sex, either. However, in women the incidence rate significantly increased from 116.5 per 100,000 person-years (CI 96.3–139.7) in S2 to 195.2 (CI 168.7–224.5) in S3, though it remained stable from S3 to S4.

**Table 2:** Main analysis: Incidence rates of stroke in the MONICA/KORA surveys S2–S4 with a baseline age of 25–74 years and a follow-up of eight years.

	S2		S3		S4	
	(1989/90–1997/98)		(1994/95–2002/03)		(1999/2001–2007/08)	
	*n* ^a^	IR^b^ (95% CI)	*n* ^a^	IR^b^ (95% CI)	*n* ^a^	IR^b^ (95% CI)
Total	79	203.4 (176.4–233.4)	81	225.6 (197.1–257.0)	65	209.9 (182.4–240.3)
Men	58	281.0 (249.1–315.9)	48	256.0 (225.6–289.4)	40	257.6 (227.1–291.0)
Women	21	116.5 (96.3–139.7)	33	195.2 (168.7–224.5)	25	169.9 (145.3–197.4)

Abbreviations: CI, confidence interval.

^a^Number of incident strokes during 8 years of follow-up.

^b^Incidence rates per 100,000 person-years. Directly age-standardized based on the German population on December 31st, 2000.

**Figure 2:**
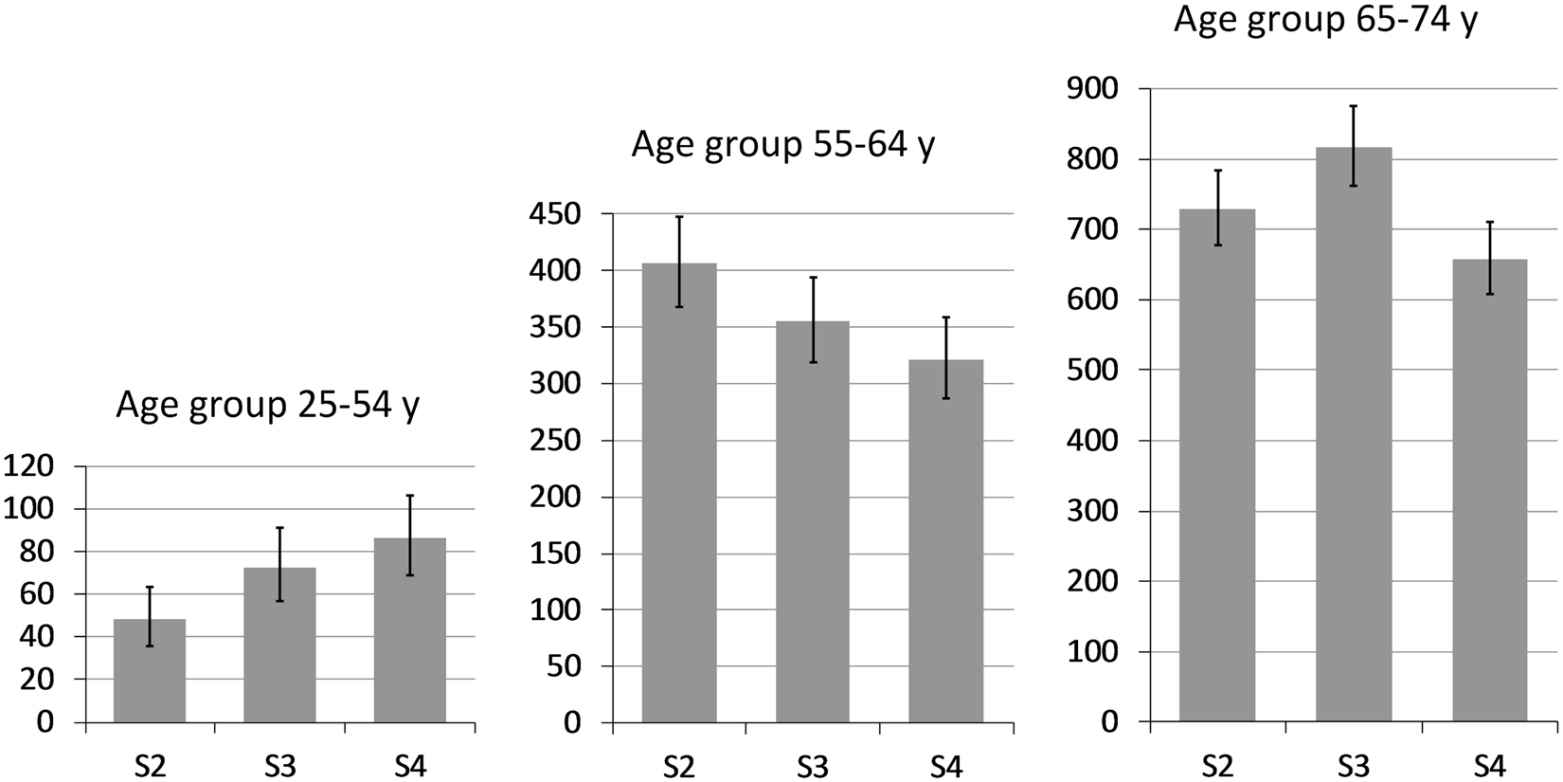
Age-standardized incidence rates of stroke with 95% confidence intervals; in the MONICA/KORA surveys S2-S4 with a baseline age of 25–74 years and a follow-up of eight years, by age group.

In order to conduct age-stratified analyses, we built 10-year age groups, but summarized the first three 10-year age groups to one group (25–54 y) because of the small number of incident stroke cases in these younger age groups. A time trend in incidence rates of strokes could not be observed in any age group (see Fig. 2). Analyses by age and sex also did not show a time trend, but a significant increase in incidence rates (IR) from S2 (IR = 497.2; CI 454.5–542.9) to S3 (IR = 763.9 CI 710.7–820.1) in women aged 65–74 y and a slightly significant increase in IR in women aged 25–54 y (S2: IR = 20.9; CI 12.9–32.0 vs. S3: IR = 44.9; CI 32.7–60.1) was present.

The sub-analysis considering only ischemic stroke confirmed the lack of a time trend in the incidence rates of stroke from S2 to S4 in participants aged 25–74 y, even when stratified by sex or by age group (see Table 3, Supplementary Table Sup1 and Supplementary Fig. Sup1).

**Table 3:** Sub-analysis: Incidence rates of ischemic stroke in the MONICA/KORA surveys S2–S4 with a baseline age of 25–74 years and a follow-up of eight years.

	S2		S3		S4	
	(1989/90–1997/98)		(1994/95–2002/03)		(1999/2001–2007/08)	
	*n* ^a^	IR^b^ (95% CI)	*n* ^a^	IR^b^ (95% CI)	*n* ^a^	IR^b^ (95% CI)
Total	62	160.5 (136.6–187.3)	63	175.6 (150.6–203.6)	53	171.2 (146.5–198.8)
Men	45	217.6 (189.7–248.5)	38	202.2 (175.3–232.1)	33	211.9 (184.4–242.5)
Women	17	95.3 (77.1–116.4)	25	148.4 (125.5–174.3)	20	136.9 (114.9–161.8)

Abbreviations: CI, confidence interval.

^a^Number of incident ischemic strokes during 8 years of follow-up.

^b^Incidence rates per 100,000 person-years. Directly age-standardized based on the German population on December 31st, 2000.

The age-standardized incidence rates of ischemic stroke in participants aged 25–64 years at baseline showed similar incidence rates for S2, S3 and S4, but distinctly lower incidence rates in S1. This was observed in the total sample as well as in men and women (see Supplementary Tables Sup1 and Sup2).

In the analysis of S2 and S3 with 14 years of follow-up, there was also no significant time trend in the incidence rates of ischemic stroke in the total population and when stratified by sex (data not shown).

The age-standardized prevalence rates of the CVD risk factors were quite stable over time: the changes between the surveys S2, S3, and S4 were small and mostly non-significant (see Table 4). Only two risk factors showed a significant time trend: the prevalence of low leisure time physical activity and of low education decreased from S2 to S4. The prevalence of current smoking, high alcohol intake and diabetes mellitus decreased from S2 to S4 as well, but these trends were not significant. The same is true for the slight increase in the prevalence of former smokers, obesity and elevated uric acid. Changes in the prevalence of the other risk factors did not consistently go in one direction. Only the fluctuation of the elevated TC/HDL ratio was significant, with an increased prevalence in S3 (versus S2) and a later decreased prevalence in S4 (versus S3). Furthermore, after a non-significant rise in the prevalence of all three hypertension-related variables from S2 to S3, the prevalence rates decreased from S3 to S4, though the change was only significant for actual hypertension and hypertension grade 2. In contrast, the percentage of antihypertensive medication slightly decreased and afterwards increased (non-significantly).

**Table 4:** Age-standardized prevalence of CVD risk factors in the MONICA/KORA Augsburg surveys S2–S4 at baseline (age 25–74 years).

	Prevalence (95% confidence interval) [%]^a^		
	S2	S3	S4
	1989/90	1994/95	1999/2001
smokers: current	27.10 (25.79–28.40)	26.52 (25.21–27.83)	26.32 (24.92–27.73)
smokers: former	27.98 (26.67–29.29)	28.22 (26.89–29.56)	31.02 (29.55–32.49)
high alcohol intake^b^	41.39 (39.94–42.84)	36.47 (35.03–37.91)	35.59 (34.05–37.13)
low physical activity^c^	60.58 (59.16–62.00)	54.74 (53.27–56.22)	49.65 (48.04–51.25)
obesity (BMI ≥ 30 kg/m²)	18.15 (17.06–19.25)	20.25 (19.09–21.42)	22.05 (20.75–23.34)
low education level^d^	73.01 (71.71–74.32)	68.31 (66.93–69.70)	59.24 (57.67–60.80)
elevated TC/HDL (≥5)	28.28 (26.97–29.59)	34.13 (32.72–35.55)	25.18 (23.80–26.56)
elevated uric acid^e^	9.60 (8.76–10.45)	10.50 (9.60–11.41)	13.58 (12.51–14.65)
actual hypertension^f^	36.20 (34.91–37.49)	37.49 (36.18–38.80)	34.40 (33.01–35.79)
hypertension (BP ≥ 160/100 mmHg)	9.89 (9.08–10.70)	10.36 (9.51–11.20)	7.26 (6.47–8.05)
hypertension (BP ≥ 180/110 mmHg)	2.11 (1.71–2.51)	2.28 (1.86–2.70)	1.67 (1.27–2.06)
antihypertensive medication	14.45 (13.55–15.34)	14.32 (13.41–15.23)	15.66 (14.65–16.67)
diabetes^g^	3.99 (3.47–4.51)	3.50 (3.00–4.01)	3.30 (2.76–3.84)
parents’ stroke^h^	19.76 (18.63–20.89)	19.37 (18.24–20.50)	20.69 (19.44–21.94)

Abbreviations: BMI, body-mass index; BP, blood pressure; TC/HDL, total cholesterol/high-density lipoprotein cholesterol ratio.

^a^Directly age-standardized based on the German population on December 31st, 2000.

^b^Alcohol intake is classified in high and moderate. High in men >20 mg/day pure alcohol, high in women >10 mg/day.

^c^No sportive activitiy in leisure time up to one hour per week irregularly.

^d^<12 years of school and apprenticeship/studies.

^e^In men >420 µmol/l, in women >360 µmol/l.

^f^BP ≥ 140/90 mmHg or intake of antihypertensive drugs (adapted from WHO/ISH Hypertension guidelines 1999).

^g^Self-reported.

^h^Mother’s and/or father’s stroke. “don’t know” is added to “no”.

Examining the sex-specific prevalence of risk factors over time revealed quite similar results (data not shown). Concerning women in the oldest age group, for which we found a significant increase in stroke incidence from S2 to S3, none of the CVD risk factors showed a significantly increased prevalence from S2 to S3. In women aged 25–54 y, only the elevated TC/HDL ratio rose significantly as the stroke incidence did from S2 to S3, while the prevalence of the other CVD risk factors remained quite stable.

## Discussion

The results of the main analyses including the surveys S2–S4 and considering the full age range of 25–74 y showed no time trend in incidence rates of age-standardized stroke over time in men and women. Only a significant increase in the incidence rate of stroke from S2 to S3 was observed in women.

In Germany, after 1990, the application of imaging methods, especially computed tomography (CT), as a standard diagnostic procedure drastically improved the quality of stroke diagnostics^16^. Thus, the distinct increase in the incidence rate of stroke from S1 to S2 seems not to derive from increased numbers of true incident cases, but rather from an increase in the number of detected cases. This phenomenon has already been described in the USA^17^.

The present results were in line with prior German studies that reported stable stroke rates in the populations from the East German MONICA stroke registers in the years 1984 to 1993 (after a preceding increase)^16,18^ and from the Erlangen stroke register in the years 1995 to 2010^19^. In contrast to our results, in men from Erlangen, the incidence of ischemic stroke decreased during the 15-year study period. However, due to the different age-ranges, neither study is fully comparable with ours. Data from Heidelberg, another German MONICA stroke register, were only published for 1985/86 and without information on time trends^20^.

In addition to the German results, studies from Dijon (France)^21^, Southern Italy^22^, and Minneapolis-St Paul (Minnesota, USA)^23^ also reported stable stroke incidence rates. A review of population-based studies from the late 20th century showed that in 7 out of 8 studies comprising populations from Finland, Japan, New Zealand and Russia, a preceding decline in first-ever stroke reached a plateau or even reversed in the late 1980s and early 1990s^5^. In the Netherlands it was shown that the incidence of age- and sex-specific first-ever ischemic stroke in people aged 35–94 years remained stable or increased slightly between 1997 and 2005^24^. However, the city of Rotterdam differs, with constant incidence rates in stroke only in women, but decreasing trends in men from 1990–2008^25^. In Sweden, diverging trends were found as well. Some studies reported stable incidence rates in stroke in both sexes from 1975–1990^26^ and from 1987–2006 in Gothenburg^27^ or at least since 2001/2002^28^. However, the MONICA register of Northern Sweden showed constant incidence rates only in women, but decreased rates in men aged 35–64 years from 1985–1991^29^. In contrast, in Southern Sweden a marked increase in the incidence of first-ever stroke was observed from 1983–1995^30^.

Results based on MONICA populations with records from stroke registers have found declining trends, though they were mostly not significant^31,32^. Further studies confirmed declining trends in stroke incidence rates in England (Oxford Vascular Study^33^ and South London Stroke Register^34^), Massachusetts, USA (Framingham successor study^35^) and Norway (Tromsø Study^6^). A systematic review by Feigin *et al*. including 56 population-based studies comprising 47 centres in 28 countries from 1970 to 2008 found a 42% decrease in stroke incidence, especially ischemic stroke, in high-income countries^4^. Similarly, recent reviews using data from the Global Burden of Diseases, Injuries, and Risk Factors Study (GBD Study) comprising 119 studies presented a significantly declining trend in the age-standardized incidence of stroke from 1990–2013 in high-income countries^3,36,37^.

In contrast, studies from low- and middle-income countries mainly reported increasing trends in stroke incidence^3,4,36^. However, as they are not comparable with our study population, they are not presented here in detail.

In the present study, the age-standardized prevalence rates of the CVD risk factors were quite stable over time. They showed diverging, but mostly non-significant changes. It is imaginable that these divergent developments neutralized each other, and therefore did not lead to changes in the incidence rate of stroke. Hence, the result of the present CVD risk factor analysis was in accordance with our finding of stable incidence rates of stroke.

The literature comprising studies from high-income countries covering the time period 1980–2010 consistently showed that results of stroke incidence trend analysis were associated with the development of the prevalence of CVD risk factors. In the East German MONICA populations, Heinemann *et al*.^38^ reported trends in CVD risk factors and Eisenblätter *et al*.^18^ were able to link them to changes in stroke incidence. The preceding rise in stroke incidence could be associated with a deteriorating risk factor profile, especially with an increase in the proportion of hypertension in men^18^. In the mid 1980s, stabilized mean cholesterol and BMI values, as well as a declining (men) or unchanged (women) prevalence of smoking were observed, in contrast to increased mean levels of systolic BP in men and slightly declined ones in women^38^. These developments are congruent with the reported stabilization of stroke trends since 1985^18^.

In the studies from Dijon (France)^21^, Southern Italy^22^, Minneapolis-St Paul (Minnesota, USA)^23^, Gothenburg (Sweden)^27^ and Rotterdam (Netherlands)^25^, which found stable incidence rates in stroke in both sexes or only in women^25^, some risk factors did not change over time, while several decreased and others increased. As in the present study, these divergent developments seemed to neutralize each other, causing a stabilization in stroke trends. Concerning hypertension results differed: stable prevalence rates were found in Italy^22^ and Minneapolis-St Paul (Minnesota, USA)^23^, decreased rates in Gothenburg (Sweden)^27^, and increased rates in women in Rotterdam (Netherlands)^25^. Harmsen *et al*. emphasized that despite decreased BP levels in Gothenburg, there was still a considerable percentage of people with hypertension, which might prohibit a decline in stroke rates^27^. The use of antihypertensive medication was stable over time in France^21^ and in women in Rotterdam, whereas it increased in Minneapolis-St Paul (Minnesota, USA)^23^.

In accordance with the reduction in stroke incidence in most of the MONICA populations, systolic BP, mean levels of serum cholesterol and the prevalence of daily cigarette smoking declined over 10 years in both sexes^7^. However, BMI increased in two-thirds of the populations in men and in half of populations in women^7^. Very similarly, declining trends in stroke rates were associated with a decrease in the prevalence rates of all of the collected classic CVD risk factors, except from an increase in BMI and/or in diabetes mellitus, in the surveys from Norway^6^, England (Oxford vascular study^33^ and South London Stroke Register^34^), and from Massachusetts, USA (Framingham successor study^35^).

The main strength of the present study is the prospective and population-based design with standardized survey methods. To the best of our knowledge, the present study is the first one to report about long-term simultaneously collected data on stroke and CVD risk factors in the same study population in Germany. The quality of the stroke occurrence data is very good due to the validation of the self-reported information. However, several limitations of the study have to be considered. Because of the lack of a stroke register, our data on stroke do not comprise all cases in the entire population of the study area, but only in study participants. Therefore, the number of stroke cases is not very high, leading to low statistical power in the analyses stratified for sex and age. Additionally, a more detailed classification of ischemic stroke into large-vessel, cardio-embolic and lacunar stroke types was not available. Moreover, smoking habits and physical activity were self-reported and may therefore be under-reported.

## Conclusions

In the region of Augsburg, Southern Germany, we found stable incidence rates in stroke over time; in parallel, the prevalence of classic CVD risk factors remained largely unchanged. However, even a stable incidence rate as also observed in several other high-income countries will lead to an increased number of total stroke cases in the population because of improved medical therapy and the growing number of old and very old adults in the population^39^. Bearing also in mind that stroke is a major cause of long-term disability, primary prevention of stroke should remain in the focus of public health activities.

## Electronic supplementary material

Supplementary information
