# Supplementary material for: Time trends in stroke incidence and in prevalence of risk factors in Southern Germany, 1989 to 2008/09
Source: Sci Rep. 2018 Aug 10;8:11981. doi: 10.1038/s41598-018-30350-8 (PMC6086828; doi:10.1038/s41598-018-30350-8)
Supplement: Supplementary file 1 — Supplementary information [file 41598_2018_30350_MOESM1_ESM.pdf]

Supplementary information about the sub-analyses

Time trends in stroke incidence and in prevalence of risk factors in Southern Germany, 1989 to 2008/09

Inke Thiele[1*], Jakob Linseisen[2,3], Margit Heier[1], Rolf Holle[4], Inge Kirchberger[3], Annette Peters[1], Barbara Thorand[1], Christa Meisinger[1,3]

[1] Institute of Epidemiology, Helmholtz Zentrum München, German Research Center for Environmental Health, Neuherberg, Germany

[2] Independent Research Group Clinical Epidemiology, Helmholtz Zentrum München, German Research Center for Environmental Health, Neuherberg, Germany

[3] Chair of Epidemiology, Ludwig-Maximilians Universität München, UNIKA-T Augsburg, Germany

[4] Institute of Health Economics and Health Care Management, Helmholtz Zentrum München, German Research Center for Environmental Health, Neuherberg, Germany

---

Table Sup1: Participants of the MONICA/KORA surveys included in the sub-analyses, including only incident ischemic stroke

	**S1** 1984/85	**S2** 1989/90	**S3** 1994/95	**S4** 1999/2001
		Participants aged 25-74 y		
total N	-	4577	4366	3747
men n (%)	-	2302 (50.29)	2166 (49.61)	1818 (48.52)
women n (%)	-	2275 (49.71)	2200 (50.39)	1929 (51.48)
		Participants aged 25-64 y		
total N	3905	3703	3588	3129
men n (%)	1966 (50.35)	1840 (49.69)	1755 (48.91)	1502 (48.00)
women n (%)	1939 (49.65)	1863 (50.31)	1833 (51.09)	1627 (52.00)

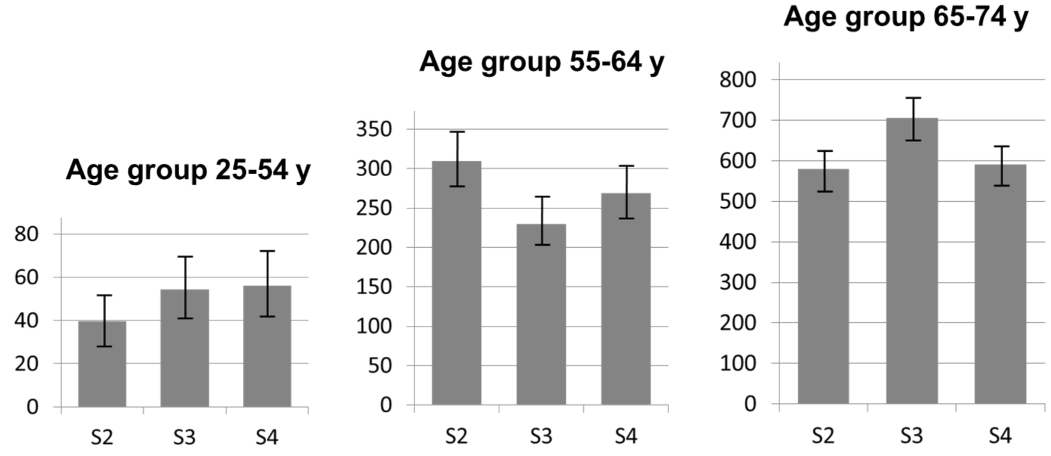

Figure Sup1: Sub-analysis: Age-standardized incidence rates of ischemic stroke in the MONICA/KORA surveys S2-S4 with a baseline age of 25-74 years and a follow-up of eight years, by age group

Table Sup2: Sub-analysis: Incidence rates of age-standardised ischemic stroke in the MONICA/KORA surveys S1-S4 with a baseline age of 25-64 years and a follow-up of eight years

	**S1** (1984/85-1992/93)		**S2** (1989/90-1997/98)		**S3** (1994/95-2002/03)		**S4** (1999/2001-2007/08)	
	n [a]	IR [b] (95% CI)	*n* [a]	IR [b] (95% CI)	*n* [a]	IR [b] (95% CI)	*n* [a]	IR [b] (95% CI)
Total	*19*	60.5 (46.2-77.8)	*30*	98.7 (80.2-120.2)	*26*	88.0 (70.6-108.4)	*27*	103.5 (84.6-125.5)
Men	*14*	84.8 (67.7-104.9)	*21*	133.6 (111.9-158.3)	*17*	116.8 (96.6-140.0)	*20*	158.9 (135.1-185.6)
Women	*5*	33.7 (23.3-47.1)	*9*	62.8 (48.2-80.4)	*9*	60.6 (46.3-77.9)	*7*	53.8 (40.4-70.2)

Abbreviations: CI, confidence interval;
a: number of incident ischemic strokes during 8 years of follow-up
b: Incidence rates per 100,000 person-years. Directly age-standardized based on the German population on December 31st, 2000
